# Enumeration of circulating endothelial cell frequency as a diagnostic marker in aortic valve surgery - a flow cytometric approach

**DOI:** 10.1186/s13019-017-0631-3

**Published:** 2017-08-09

**Authors:** Anton Sabashnikov, Klaus Neef, Vera Chesnokova, Leonie Wegener, Kathrin Godthardt, Maximilian Scherner, Elmar W. Kuhn, Antje-Christin Deppe, Meike Lauer, Kaveh Eghbalzadeh, Mohamed Zeriouh, Parwis B. Rahmanian, Jens Wippermann, Ferdinand Kuhn-Régnier, Navid Madershahian, Thorsten Wahlers, Alexander Weymann, Yeong-Hoon Choi

**Affiliations:** 1 Department of Cardiothoracic Surgery, Heart Center, University Hospital Cologne, Cologne, Germany; 2 Center for Molecular Medicine Cologne, University Hospital Cologne, Cologne, Germany; 3 Miltenyi Biotec GmbH, Bergisch-Gladbach, Germany; 4 Department of Cardiac Surgery, European Medical School Oldenburg-Groningen, Carl von Ossietzky University Oldenburg, Oldenburg, Germany

**Keywords:** Aortic valve surgery, Circulating endothelial cells, Flow cytometry

## Abstract

**Background:**

The frequency of circulating endothelial cells (CEC) in patients’ peripheral blood can be assessed as a direct marker of endothelial damage. However, conventional enumeration methods are extremely challenging. We developed a novel, automated approach to determine CEC frequencies and tested this method on two groups of patients undergoing conventional (CAVR) versus trans-catheter aortic valve implantation (TAVI).

**Methods:**

CEC frequencies were assessed by a flow cytometric approach, including automated pre-enrichment of CD34 positive blood cell subpopulation and isotype controls. The efficacy and reproducibility of the CEC enumeration method was validated by spiking blood samples of healthy control donors with defined numbers of endothelial cells.

**Results:**

CEC frequencies were significantly higher in the TAVI group before (9.8 ± 4.1 vs. 5.5 ± 2.2, *p* = 0.019) and 1 h after surgery (13.4 ± 5.1 vs. 8.2 ± 4.1, *p* = 0.030) corresponding to higher Euroscore, STS score in higher risk patients from the TAVI group. Five days after surgery, CEC frequencies became significantly higher in the more invasive CAVR group (39.0 ± 13.0 vs. 14.3 ± 4.4, *p* < 0.001) compared to minimally invasive TAVI approach.

**Conclusions:**

The new flow cytometric approach might be a robust and reliable method for CEC enumeration. Initial results show that CEC frequency is a valid clinical marker for the assessment of pre-operative risk, invasiveness of surgical procedure and clinical outcome. Further studies are necessary to validate the practical clinical usefulness and the potential superiority compared to conventional markers.

## Background

Endothelial cells regulate the interaction between components of the blood stream and the vascularized tissue. Endothelial injury compromises these functions resulting in increased thrombogenicity and induction of the inflammatory cascade [1]. Endothelial damage typically arises from acute injury or chronic disease and is traditionally diagnosed by secreted molecule markers in the blood stream, i.e. von-Willebrand factor and thrombomodulin. However, plasma concentrations of these markers are subject to intra-patient variability and may be affected by confounders such as concomitant procedures [2–4]. The detection of mature endothelial cells in the peripheral blood has emerged as an alternative marker for endothelial injury [5, 6]. These cells are detached from the vessel walls as an immediate consequence of compromised endothelial integrity and can be detected as CEC in the peripheral blood [7]. The rarity of the indicative event, i.e. low frequency of CEC within the multitude of other blood cells, explains the need for the robustness and reproducibility of the detection method. The original method for quantification of CEC was based on the immuno-magnetic enrichment of CEC (CD146 or CD34), followed by manual microscopic enumeration assessment of the labeled cells [7]. Although standardization of this procedure has been proposed [8], it suffers significantly from variable cell loss during manual cell isolation, the need for error-prone visual microscopic screening and the lack of rigid controls and documentation. Flow cytometric approaches were introduced to address this inaccuracy by enabling high throughput measurements of cell suspensions with negative controls by using non-specific isotype antibodies [9, 10]. In order to improve the robustness and the reproducibility of this approach the total number of events measured was reduced by immuno-magnetic pre-sorting of blood cell sub-populations prior to flow cytometric analysis [11], which has also been carried out semi-automatically on a platform originally designed for the detection of circulating cancer cells [12, 13].

For healthy persons and under physiological conditions CEC are present at very low concentration [14]. Increased levels of CEC have been shown for a variety of severe diseases and conditions, such as acute coronary syndrome [14], acute infections [15–17], chronic renal failure [18], malignant tumors [19], diabetes mellitus type I [20] and II [21], peripheral arterial occlusive disease [22], vasculitides [23], sickle cell anemia [24], thrombotic microangiopathy [25], chronic venous insufficiency [26] and after allogeneic hematopoietic progenitor cell transplantation [27]. In the field of cardiovascular disease CEC have recently been shown as a marker of endothelial injury with clinical relevance in cases of acute myocardial infarction [28, 29]. Furthermore, the detachment of endothelial cells into the blood stream represents one of the multiple severe adverse effects of CPB [30–33] that is required for most cardiac surgical procedures.

CAVR represents a routine surgical procedure that involves median sternotomy as a mean to access the operational situs and CPB. TAVI procedure was initially introduced for the treatment of severe aortic valve stenosis in high-risk patients as well as in patients with contraindications for surgery. This minimally invasive approach underwent an extensive development over the last decade [34, 35]. Despite acceptable postoperative morbidity and mortality of high risk TAVI patients [34], this surgery may be associated with an increased number of technical complications, such as incorrect positioning leading to displacement, paravalvular leaks or partial coronary occlusion and, therefore, has not been recommended for the treatment of low risk patients who can tolerate a more invasive conventional procedure [35].

Our new robust method for the assessment of CEC frequency as a novel parameter of endothelial integrity is suggested to be a valuable and reliable tool in assessing both, the pre-operative risk level determined by present comorbidities and the extent of perioperative inflammation triggered by the use of CPB. We applied an optimized and automated method for the detection and enumeration of CEC in the peripheral blood and included two patient cohorts with conceivable differences in pre-operative condition, with respect to comorbidities and age, resulting in distinct conventionally designated pre-operative risk scores. The rationale for choosing these different patient cohorts for the assessment of CEC frequency was to test the validity of this novel marker of endothelial damage by comparing these two patient cohorts (details in the Results and Discussion sections). The second rationale and the ultimate aim of this study was to demonstrate the clinical relevance of CEC as a marker of endothelial damage with potential diagnostic and prognostic value for patients with severe aortic valve stenosis undergoing either minimally invasive procedure (TAVI) or conventional technique using CPB (CAVR).

## Methods

### Circulating endothelial cell enumeration

CEC levels were determined and analyzed at 4 time points perioperatively: immediately before skin incision, 1 h postoperatively, on day 1 and day 5 after surgery. The enumeration of CEC in peripheral blood was performed using the CEC enrichment and enumeration kit (Miltenyi Biotec, Bergisch-Gladbach, Germany) according to the manufacturer’s instructions. Briefly, the determination of CEC frequency is based on the flow cytometric detection and quantification using a MACSquant flow cytometer (Miltenyi Biotec) equipped with 488 nm and 635 nm laser for fluorescence excitation. For the characterization of cells the following antibodies (with fluorescence label) were included in the kit: anti-CD34 (fluorescein isothiocyanate, FITC), anti-CD146 (allophycocyanin, APC), anti-CD45 (R-phycoerythrin, PE). After excluding cell debris using the forward scatter (FSC) and sideward scatter (SSC), cells positive for CD34 and CD146, while negative for CD45 and propidium iodide (PI, as a positive marker of dead cells) were considered CEC. In order to enhance the accuracy of detecting these low-frequency target cells, the blood sample was enriched prior to analysis by red blood cell lysis and automated magnetic cell sorting of CD34 expressing cells (via anti-CD34 antibodies coupled to superparamagnetic microparticles, included in the kit) using the internal MACSQuant column of the MACSquant flow cytometer. Blood samples of patients were acquired at indicated time points using EDTA-anticoagulant collection tubes (Sarstedt, Nümbrecht, Germany) and were stored for a maximum of 12 h at 4 °C. A total sample volume of 10.2 mL was used for the CEC enumeration procedure. Each blood sample was split into three fractions, which were processed individually: 1) original fraction (0.2 mL), 2) CEC staining fraction (5 mL) and 3) CEC isotype control fraction (5 mL). The original fraction served as control for the quality of the blood sample and frequency of CD34 positive cells before enrichment. The CEC staining sample served for the actual CEC detection in a pre-enriched cell fraction applying the indicated target cell characteristics. The CEC isotype sample served as a control for the detection specificity in a pre-enriched cell fraction by exchanging the specific anti-CD146 antibody with an isotype matched unspecific antibody.

In order to validate the efficacy and reproducibility of the CEC enumeration method blood samples of healthy donors were spiked with defined numbers of endothelial cells.

### Human arterial endothelial cell isolation

LIMA samples were obtained from residual material of vessels prepared in elective patients undergoing CABG at the Department of Cardiothoracic Surgery at the University Hospital Cologne. Informed consent was obtained from the patients prior to surgery and this analysis was conducted upon approval by the local ethics committee. The tissue was rinsed with PBS (Invitrogen, Karlsruhe, Germany) and clamped at one end. A solution of 2 mg/mL collagenase type I (Invitrogen, Darmstadt, Germany) in HBBS (Invitrogen) was injected into the lumen and the tissue was incubated at 37 °C for 15 min. The clamp was then removed and the lumen flushed with HBSS to collect the endothelial cells. Cells were washed twice with HBSS and counted using a hemacytometer (Roth, Karlsruhe, Germany).

### Patients

In order to test the new approach we also prospectively analyzed a cohort of patients assigned to either CAVR or TAVI. This study was approved by the Institutional Ethics Committee and individual informed consent was obtained from each patient included. Twenty-two consecutive patients with severe aortic valve stenosis scheduled for isolated elective aortic valve replacement surgery were enrolled in the study and assigned to either CAVR (*n* = 11) or TAVI (*n* = 11) group. The assignment of the patients to each group was performed by our institutional interdisciplinary heart team consisting of at least two cardiac surgeons and two cardiologists. The main criterion for the patients with aortic valve stenosis to be considered for TAVI procedure was the unacceptably high predicted risk associated with conventional surgery based on age, Euroscore and presence of comorbidities.

The exclusion criteria were the presence of the above-mentioned diseases and conditions, primarily leading to increased levels of CEC, as well as urgent (acute onset or deterioration of potentially life-threatening condition) and emergent (immediate life-saving procedures) cases. Perioperative data were collected in the same time period. CEC as well as serology and further perioperative clinical end points (in-hospital mortality, hospital and intensive care unit stay, ventilation time, blood loss, transfusion requirements, post-operative atrial fibrillation, renal insufficiency, neurocognitive disturbance etc.) were assessed by evaluating patients’ medical notes during their hospital stay and after discharge.

### Surgical procedure and peri-operative care

All patients underwent regulated pre-operative evaluation including structured interview, medical examination, laboratory assessment, ECG, chest X-ray examination, TTE and TEE, lung function test and coronary angiography. Perioperative evaluation of TAVI patients included additional CTA of the heart, the thoraco-abdominal aorta as well as the iliac and femoral arteries. TAVI procedures were performed in cooperation with interventional cardiologists and CAVR were performed by senior cardiac surgeons in cooperation with experienced anesthesiologists and perfusionists. In all TAVI cases a senior perfusionist was involved stand by for potential emergent conversion to sternotomy and CPB.

### Conventional aortic valve replacement

The surgical access for conventional surgery was standard median sternotomy. In all cases 300 IU/kg body weight of heparin were administered prior to establishment of CPB with an arterial cannula in the ascending aorta and a two-stage venous cannula introduced through the right atrium.

### Trans-catheter aortic valve implantation

The TAVI procedures were performed in the hybrid operating room of our department using standard approaches previously described in the literature [35]. Our patients underwent TAVI either in the antegrade way using direct trans-apical access (*n* = 9) or in the retrograde way using trans-femoral approach (*n* = 2). In either case the native aortic valve was predilated by an expandable valvuloplasty balloon during the rapid ventricular pacing. The prosthetic valve was positioned and deployed once the satisfactory position was achieved. Accurate positioning as well as prosthetic valve function was checked using trans-oesophageal echocardiography and contrast aortography. In all cases 150 IU/kg body weight of heparin were administered for sufficient anticoagulation. Postoperatively, all patients were transferred for further observation to the ICU of our department. Perioperative care of the patients was performed according to internal standard protocols.

### Statistical analysis

Statistical analysis was performed using IBM SPSS Statistics version 21 software (SPSS, Chicago, IL). All continuous variables were expressed as mean ± standard deviation. Categorical data were expressed as total numbers and percentages. Pearson’s χ2 or Fisher’s exact tests were used for categorical data depending on the minimum expected count in each cross tabulation. Statistical analysis of metric parameters was carried out using the Student *t*-test. A *p*-value of <0.05 was considered to indicate statistical significance.

## Results

### Demographic data

Table 1 shows patients’ demographic data, comorbidities and preoperative clinical characteristics for CAVR and TAVI groups. TAVI patients were significantly older, had higher Euroscore and STS score, higher serum creatinine levels, higher incidence of pre-operative cardiac decompensation, lower hemoglobin concentration, lower height and lower body weight compared to CAVR patients. History of CAD, arterial hypertension, hyperlipidemia, and history of myocardial infarction were equally distributed between both groups. There were no considerable discrepancies in terms of gender distribution, left ventricular ejection fraction as well as aortic valve area.

**Table 1:** Patient demographics and perioperative data

	CAVR (*n* = 11)	TAVI (*n* = 11)	*p*-value
Age [yrs]	69.0 ± 11.2	81.0 ± 4.8	**0.004**
Female [N]	5 (45.5%)	7 (63.6%)	0.670
Height [cm]	175.6 ± 5.0	166.2 ± 9.2	**0.011**
Weight [kg]	84.9 ± 11.6	65.5 ± 12.4	**0.002**
Log. euroSCORE	5.33 ± 4.40	19.37 ± 7.05	**<0.001**
STS score	1.90 ± 1.06	7.85 ± 3.59	**<0.001**
CAD [N]	4 (36.4%)	8 (72.7%)	0.198
Pre-operative creatinine [mg/dL]	0.92 ± 0.2	1.52 ± 0.9	**0.042**
Pre-operative hemoglobin [g/dL]	13.8 ± 1.3	11.6 ± 0.9	**0.001**
Pre-operative CK [U/L]	63.8 ± 24.7	42.0 ± 27.1	0.053
Pre-operative CK-MB [U/L]	6.73 ± 1.6	10.36 ± 5.6	0.053
Pre-operative Troponin T [μg/L]	0.01 ± 0.01	0.08 ± 0.10	**0.031**
Pre-operative EF [%]	61.9 ± 23.1	49.9 ± 26.0	0.355
Pre-operative AVA [cm^2^]	0.62 ± 0.30	0.60 ± 0.22	0.922
Pre-operative cardiac decompensation [N]	0	4 (36.4%)	**0.045**
Arterial hypertension [N]	10 (90.9%)	11 (100.0%)	1.000
Hyperlipoproteinemia [N]	10 (90.9%)	11 (100.0%)	1.000
Pre-operative pulmonary hypertension [N]	0	2 (18.2%)	0.476
Previous statin therapy [N]	10 (90.9%)	11 (100.0%)	1.000
History of AMI [N]	0	1 (9.1%)	0.168

*AVA* aortic valve area, *CAD* coronary artery disease, *CK* creatine kinase, *CK-MB* creatine kinase-muscle/brain (cardiac specific isoform), *EF* ejection fraction, *AMI* acute myocardial infarction

Statistically significant *p*-values are presented in bold

### Peri-operative data and outcome

**Fig. 1:**
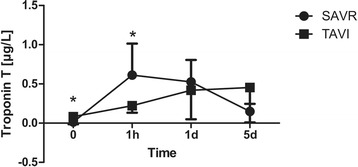
Cardiac enzymes peri-operatively: Troponin T. Serial time course of Troponin T levels in patients operated using CAVR or TAVI technique. Troponin T levels were significantly higher in the TAVI group pre-operatively. One hour after surgery, however, Troponin T levels became significantly higher in the CAVR group. There were no significant differences during further post-operative course (*p < 0.05, **p < 0.001)

**Fig. 2:**
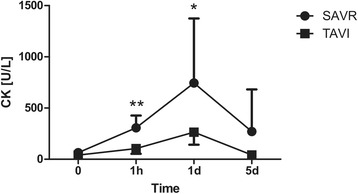
Cardiac enzymes peri-operatively: CK. Serial time course of CK levels in patients operated using CAVR or TAVI technique. CK levels were comparable pre-operatively and significantly higher 1 h and 1 d after surgery in the CAVR group compared to the TAVI group. This difference did not reach statistical significance 5 d after surgery (*p < 0.05, **p < 0.001)

**Fig. 3:**
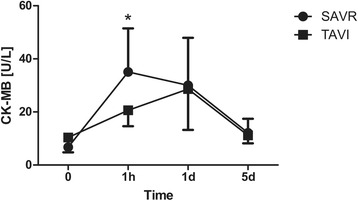
Cardiac enzymes peri-operatively: CK-MB. Serial time course of CK-MB levels in patients operated using CAVR or TAVI technique. CK-MB levels were comparable pre-operatively and significantly higher 1 h after surgery in the CAVR group. There were no statistically significant differences between the two groups during further post-operative course. (*p < 0.05, **p < 0.001)

**Fig. 4:**
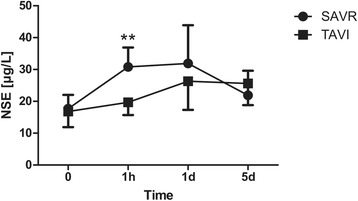
Neuron specific enolase (NSE) peri-operatively. Serial time course of NSE levels in patients operated using CAVR or TAVI technique. NSE levels were significantly higher 1 h after surgery in the CAVR group and were comparable in both groups pre-operatively as well as 1 d and 5 d after surgery (*p < 0.05, **p < 0.001)

The operating time as well as the total chest tube drainage were higher in the CAVR group (Tables 2 and 3). There were no significant differences regarding duration of total hospital and ICU stay, transfusion requirements, incidence of post-operative neurocognitive disturbance, atrial fibrillation and requirement for dialysis. The mean post-operative peak aortic valve gradient was significantly higher in CAVR patients compared to TAVI group. Additionally, the serum levels of CK (CAVR: 306.9 ± 120.1 vs. TAVI: 104.2 ± 51.5 U/L, *p* < 0.001), CK-MB (CAVR: 35.1 ± 16.4 vs. TAVI: 20.6 ± 6.0 U/L, *p* = 0.012), Troponin T (CAVR: 0.613 ± 0.402 vs. TAVI: 0.223 ± 0.090 μg/L, *p* = 0.031) and NSE (CAVR: 30.9 ± 6.1 vs TAVI: 19.7 ± 4.0 μg/L, *p* < 0.001) were significantly higher 1 h after surgery in the CAVR group (Figs. 1, 2, 3 and 4). There were no cases of cerebrovascular accident observed in either group. In all TAVI cases there was no need for conversion to CPB or median sternotomy for CAVR. One single patient in the TAVI group died on 18th post-operative day due to pneumonia resulting in severe sepsis and multi-organ failure.

**Table 2:** Intra-operative data

	CAVR	TAVI	*p*-Wert
Operating time [min]	152.9 ± 45.9	74.2 ± 9.1	**<0.001**
Transfusion of PRBC [units]	0.6 ± 1.3	1.1 ± 1.0	0.97
Transfusion of FFP [units]	0.6 ± 1.3	0	0.177
Transfusion of platelets [units]	0.9 ± 0.3	0	0.329
CPB [min]	86.9 ± 20.5	n.a.	n.a.
Aortic Cross-clamp time [min]	57.0 ± 15.6	n.a.	n.a.
Reperfusion time [min]	20.0 ± 7.0	n.a.	n.a.

*FFP* fresh frozen plasma, *PRBC* packed red blood cells, *CPB* cardio-pulmonary bypass, *n.a.* not applicable

Statistically significant *p*-values are presented in bold

**Table 3:** Post-operative data

	CAVR	TAVI	p-Wert
Ventilation time [min]	7.6 ± 1.8	8.4 ± 0.4	0.256
Total chest tube dranaige [mL]	880.0 ± 453.0	523.0 ± 152.7	**0.048**
ICU stay [d]	3.0 ± 2.2	5.9 ± 5.6	0.126
In-hospital stay [d]	13.6 ± 5.3	14.4 ± 6.5	0.750
Transfusion of PRBC [units]	1.4 ± 1.8	1.6 ± 1.7	0.724
Transfusion of FFP [units]	0.8 ± 1.8	0.5 ± 0.9	0.564
Transfusion of platetets [units]	0.1 ± 0.3	0	0.564
Paravalvular leak [N]	0	3 (27.3%)	0.214
P_max_ [mm Hg]	25.6 ± 12.9	15.8 ± 6.3	**0.044**
P_mean_ [mm Hg]	14.6 ± 9.5	8.4 ± 4.1	0.194
V_max_ [cm/s]	285.2 ± 61.3	248.5 ± 118.9	0.517
Symptomatic transitory psychotic syndrome	1 (9.1%)	5 (45.5%)	0.149
Atrial fibrillation [N]	7 (63.6%)	10 (90.9%)	0.311
Use of class III antiarrhythmics [N]	6 (54.5%)	8 (72.7%)	0.659
Readmission to the ICU [N]	1 (9.1%)	1 (9.1%)	1.000
Reintubation [N]	1 (9.1%)	0	1.000
Sepsis [N]	0	1 (9.1%)	1.000
Creatinine 24–48 h after surgery [mg/dL]	1.4 ± 0.9	1.9 ± 1.2	0.322
CVVH [N]	2 (18.2%)	2 (18.2%)	1.000
Wound infection [N]	0	2 (18.2%)	0.476
Cerebral vascular accident [N]	0	0	
Hospital mortality [N]	0	1 (9.1%)	1.000

*CVVH* continuous veno-venous hemodialysis, *FFP* fresh frozen plasma, *ICU* intensive care unit, *P*_*max*_ maximum pressure gradient, *P*_*mean*_ mean pressure gradient, *V*_*max*_ maximum velocity, *PRBC* packed red blood cells

Statistically significant *p*-values are presented in bold

### Circulating endothelial cell concentration and clinical impact

The efficacy of CEC quantification was validated by measuring the frequency of endothelial cells in blood samples that were beforehand spiked with a defined number of freshly prepared coronary artery endothelial cells. Here, the recovery (detected vs. spiked cells) in a range of 50 to 200 endothelial cells /mL showed a significant correlation (*p* = 0.001; R [2] = 0.617; *n* = 4).

**Fig. 5:**
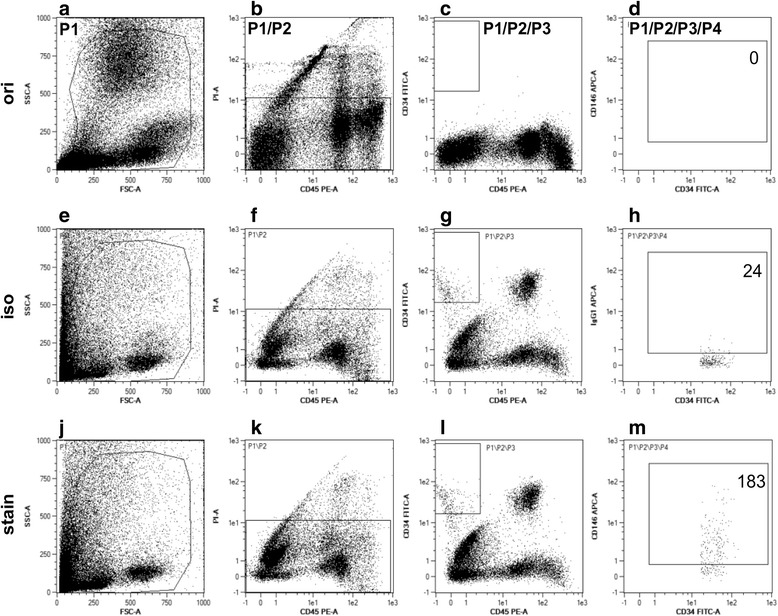
Flow cytometric detection and enumeration of CEC from peripheral blood**.** CEC frequency was assessed via a composite analysis, consisting of three separate flow cytometric analyses of a single blood sample after erythrocyte lysis. **a-d:** ori sample, not immuno-magnetically enriched; **e-h:** iso sample, immuno-magnetically enriched for CD34+ cells, antibody for CEC indicative CD146+ staining exchanged with isotype matched unspecific antibody; **j-m:** stain sample, immuno-magnetically enriched for CD34+ cells, with specific antibody for CEC indicative CD146+ staining. **a, e, j:** Dot plots for exclusion of debris and remaining erythrocytes (x-axis: forward scatter FCS; y-axis: sideward scatter, SSC) resulting in gate P1. **b, f, k:** Dot plots for exclusion of dead cells (x-axis: CD45; y-axis: propidium iodide, PI) gated in P1 resulting in gate P2 (P1/P2). **c, g, l:** Dot plots for exclusion of hematopoietic cells (x-axis: CD45; y-axis: CD34) gated in P2 resulting in gate P3 (P1/P2/P3). **d, h, m:** Dot plots for validation of CEC (x-axis: CD34; y-axis: CD146) gated in P3 resulting in gate P4 (P1/P2/P3/P4). The number displayed in the P4 gate represents the absolute number of detected CEC in the respective fraction of the blood sample (ori: 100 μl; iso: 5 ml; stain: 5 ml)

During the enumeration process the frequency of CD34^+^ cells was typically increased by 1000×, when comparing the original fraction (0.01% CD34^+^ cells within vital cell fraction) with the enriched CEC staining sample (10% CD34^+^) and the background of false positive events was below 15%, comparing the enriched CEC staining fraction with the enriched CEC isotype sample (Fig. 5).

**Fig. 6:**
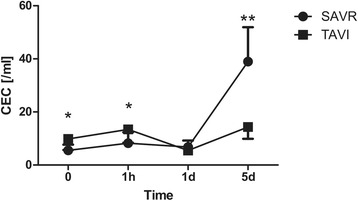
Peri-operative numbers of CEC. Serial time course of CEC frequency in patients operated on using CAVR and TAVI techniques. CEC frequency was significantly higher in the TAVI group pre-operatively as well as 1 h after surgery. On the fifth post-operative day CEC frequency was statistically higher in the CAVR group (*p < 0.05, **p < 0.001)

Regarding the CEC frequencies in the peripheral blood of TAVI and CAVR patients, pre-operatively acquired samples revealed significant higher CEC in the TAVI group (9.8 ± 4.1 vs. 5.5 ± 2.2 /mL, *p* = 0.019) as well as 1 h after surgery (13.4 ± 5.1 vs. 8.2 ± 4.1 /mL, *p* = 0.030). On 5th post-operative day there were a statistically significant higher CEC frequency in the CAVR group (39.0 ± 13.0 vs. 14.3 ± 4.4 /mL, *p* < 0.001) compared to TAVI (Fig. 6).

Despite small patient cohort observed in this trial we were able to find similarities between CEC levels and a number of clinical parameters routinely used for preoperative risk assessment and postoperative course. Our analysis showed that similarly to higher pre-operative Euroscore (TAVI: 19.37 ± 7.05 vs. CAVR: 5.33 ± 4.40; *p* < 0.001) and pre-operative STS score (TAVI: 7.85 ± 3.59 vs. CAVR: 1.90 ± 1.06; *p* < 0.001), CEC levels (TAVI: 9.81 ± 4.14 /mL vs. CAVR: 5.53 ± 2.16 /mL; *p* = 0.019) were also in consistence with a higher pre-operative risk in TAVI patients compared to CAVR patients.

Additionally, all patients with pre-operative CEC levels higher than 10/mL (*n* = 5, mean: 13.06 ± 1.78 /mL) had past medical history of CAD, developed post-operative atrial fibrillation during the post-operative course and tended to have higher post-operative creatinine levels (2.0 ± 1.3 vs. 1.2 ± 0.2 mg/dL, *p* = 0.066), prolonged ICU stay (7.2 ± 7.4 vs. 2.4 ± 0.7 days, *p* = 0.034) and higher rate of transitory psychotic disorder (80% vs. 0%, *p* = 0.002). No cases of CAD and transitory psychotic disorders were found in the subgroup of patients with CEC levels less than 5 /mL (*n* = 6). Additionally, those patients showed a trend towards lower post-operative creatinine levels (1.19 ± 0.25 vs. 1.6 ± 0.92 mg/dL) and shorter ICU stay (2.5 ± 0.84 vs. 4.6 ± 5.3 days), however not reaching statistical significance.

## Discussion

The need for new and reliable markers of endothelial damage has attracted several research groups in exploiting the potential diagnostic and prognostic value of CEC frequency in human blood. Since CEC have been proposed as a valuable clinical marker, the accurate quantitative assessment of CEC frequency using standardized and reproducible methods has become an essential requirement. Especially, research in the fields of cancer [36, 37] and cardiovascular diseases [28, 29, 38] have used various methods for the assessment of CEC frequency to investigate its correlation with clinical diagnostics and outcome. Here, notable differences have been reported for CEC frequencies in control samples from healthy human donors ranging from 4 to 1300 cells per ml of peripheral blood, depending on the method applied [11, 39–41].

Taking these aspects into consideration, the overall objective of this study was to adopt a new reliable and reproducible method of CEC enumeration and to demonstrate the clinical relevance of CEC measured by this approach in different patient cohorts. The presented method for CEC identification and enumeration is characterized by two main aspects increasing accuracy and reproducibility. Firstly, the immuno-magnetic pre-enrichment of a CD34^+^ sub-population of cells from peripheral blood and secondly, the flow cytometric detection and enumeration (absolute cell number per sample) of CEC using an integrated workflow. This method is similar to the one described before using a CellTracks system, with the main difference of making use of the CD34^+^ and not CD146^+^ antigen for immuno-magnetic separation [12]. This variation has the advantage of initial exclusion of contaminating CD146^+^ activated lymphocyte subset [42], which were shown to be responsible for over-estimation of CEC numbers in earlier reports [43, 44]. In order to document the robustness and overall validity to of the method applied, we performed spiking experiments with isolated mature endothelial cells, simulating as close as possible the actual in vivo situation of CEC, and could show a significant correlation between recovered and spiked cells. Analyzing two groups of patients with clearly different preoperative clinical state with respect to co-morbidities and age on one hand and different invasiveness of the procedure performed on the other served as a secondary control of the validity of our methodological approach. Again, our results confirmed higher preoperative endothelial damage for TAVI patients as expressed by significantly higher preoperative CEC frequencies as compared to patients receiving conventional CAVR treatment. On the other hand, CAVR patients were associated with significantly higher systemic inflammation compared to the TAVI group after more invasive surgery. Thus, investigation of two different cohorts of patients operated on conventionally and using minimally invasive approach, revealed the importance of CEC as an alternative and direct method of quantitative assessment of pre-operative risk as well as post-operative systemic injury level. Also, it could be shown that patients undergoing CAVR procedure suffered from several complications underlined by higher post-operative CEC-values compared to the TAVI group, though those patients were significantly younger, with a lower Euroscore and STS score and had fewer comorbidities. This finding was expected and corroborates previous research suggesting that CEC seem to be a direct quantitative parameter of endothelial injury caused by the CPB which leads to systemic and organ damage [31, 33].

In reviewing the literature, a great deal of data on the association between CEC and different clinical parameters have been described [14, 19, 20, 23, 25, 45]. Supporting these previous results we were able to show a strong relationship between CEC and different clinical variables with the view to revealing the diagnostic role of CEC during the post-operative course.

First, we demonstrated statistically increased preoperative CEC levels in the TAVI group consisting of elderly patients with higher Euroscore, which is currently in daily use in cardiac surgery [46], higher creatinine levels and higher incidence of cardiac decompensations as compared to the CAVR group, though all the patients with diseases known to have elevated CEC levels were excluded from this clinical trial. This correlation of CEC with increased surgical risk demonstrated by using our novel CEC enumeration approach has not previously been described. Hence, it could conceivably be hypothesized that CEC may be a major predictor of the perioperative risk of patients admitted either for conventional or minimally invasive surgical procedures. Additionally, particularly as the aggressiveness of indication for TAVI has been significantly progressing over the last decade, CEC might help clinicians in making decisions as to which surgical approach should be considered as a treatment.

Second, one of the most striking observations to emerge from the data comparison were statistically higher post-operative CEC levels in the CAVR group, emphasizing adverse effects of CPB and more invasive surgical access. This elevation is even consolidated by the dilution effect in the CAVR group caused by the priming solution required for the circuit and is delivered into patients’ circulation once CPB is connected and started. However, the postoperative difference in CEC frequency between the CAVR and TAVI groups only became significant after a latent phase on the fifth post-operative day. This interesting finding might be explained in many ways. First, in addition to the general deleterious effects of CPB the observed increase in CEC over the post-operative course could be attributed to the various complications and side effects of both surgical approaches prevailing in the CAVR group. This theory can be supported by the fact that individual patients with severe post-operative adverse events such as atrial fibrillation or acute renal insufficiency were demonstrated to have remarkably significant elevations in CEC suggesting a strong correlation between CEC and the above mentioned clinical manifestations. Second, the late phase of the CEC rise might be a response to the specific post-operative treatment regimen of patients operated on using CPB, such as significantly longer operating time (Table 2) more intensive inotropic support, stronger anticoagulation, more aggressive chest tube drainage (Table 3) and diuretic therapy. Finally, more invasive access with median sternotomy and aortotomy, use of cardioplegia and induced cardiac arrest in the CAVR group may have additional impact on the detachment of endothelial cells. Similarly to pre-operative findings, the obvious relation between CEC and various clinical parameters can be emphasized by signifikantly higher levels of different serologic parameters during the post-operative course in the CAVR group, such as CK, CK-MB, Troponin T and NSE (Figs. 1, 2, 3 and 6).

### Limitations of the study

The clinical part of the present study was designed as a single center trial with a small patient sample size in order to provide initial results using the new CEC enumeration approach. Due to the small patient cohort, no statistical correlation analysis of Euroscore, STS score and CEC was performed and statistical results should be interpreted with caution. Therefore, the findings may not be transferable to the general cohort. At last, there were no long-term results obtained and, in turn, larger clinical trials are required to confirm the present preliminary results.

## Conclusions

Our initial results showed that CEC frequency could be used as a predictive and diagnostic marker in cardiac surgical procedures, such as AVR. The new flow cytometric was shown to be an easily performable and reliable approach of CEC enumeration. However, further larger studies are necessary to confirm its clinical value as well as potential superiority compared to conventional markers.

## Abbreviations

AVR: Aortic valve replacement
CABG: Coronary artery bypass grafting
CAD: Coronary artery disease
CAVR: Conventional aortic valve replacement
CEC: Circulating endothelial cells
CK: Creatine kinase
CK-MB: Creatine kinase-muscle/brain (cardiac specific isoform)
CPB: Cardio-pulmonary bypass
CTA: Computer tomographic angiography
ECG: Electrocardiogram
HBBS: Hank’s Buffered Saline Solution
ICU: Intensive care unit
LIMA: Left internal mammary artery
NSE: Neuron specific enolase
PBS: Phosphate buffered saline
TAVI: Trans-catheter aortic valve implantation
TEE: Trans-esophageal echocardiography
TTE: Trans-thoracic echocardiography

## References

[1] Cines DB, Pollak ES, Buck CA, Loscalzo J, Zimmerman GA, McEver RP, Pober JS, Wick TM, Konkle BA, Schwartz BS, Barnathan ES, McCrae KR, Hug BA, Schmidt AM, Stern DM (1998). Endothelial cells in physiology and in the pathophysiology of vascular disorders. Blood.

[2] Horvath B, Hegedus D, Szapary L, Marton Z, Alexy T, Koltai K, Czopf L, Wittmann I, Juricskay I, Toth K, Kesmarky G (2004). Measurement of von Willebrand factor as the marker of endothelial dysfunction in vascular diseases. Exp Clin Cardiol.

[3] Lip GY, Blann A (1997). von Willebrand factor: a marker of endothelial dysfunction in vascular disorders?. Cardiovasc Res.

[4] Takahashi H, Ito S, Hanano M, Wada K, Niwano H, Seki Y, Shibata A (1992). Circulating thrombomodulin as a novel endothelial cell marker: comparison of its behavior with von Willebrand factor and tissue-type plasminogen activator. Am J Hematol.

[5] Fadini GP, Avogaro A (2010). Cell-based methods for ex vivo evaluation of human endothelial biology. Cardiovasc Res.

[6] Shantsila E, Blann AD, Lip GY (2008). Circulating endothelial cells: from bench to clinical practice. J Thromb Haemost.

[7] George F, Brisson C, Poncelet P, Laurent JC, Massot O, Arnoux D, Ambrosi P, Klein-Soyer C, Cazenave JP, Sampol J (1992). Rapid isolation of human endothelial cells from whole blood using S-Endo1 monoclonal antibody coupled to immuno-magnetic beads: demonstration of endothelial injury after angioplasty. Thromb Haemost.

[8] Woywodt A, Blann AD, Kirsch T, Erdbruegger U, Banzet N, Haubitz M, Dignat-George F (2006). Isolation and enumeration of circulating endothelial cells by immunomagnetic isolation: proposal of a definition and a consensus protocol. J Thromb Haemost.

[9] Dignat-George F (2006). Detection of circulating endothelial cells and endothelial progenitor cells by flow cytometry. Cytometry B Clin Cytom.

[10] Khan SS, Solomon MA, McCoy JP (2005). Detection of circulating endothelial cells and endothelial progenitor cells by flow cytometry. Cytometry B Clin Cytom.

[11] Widemann A, Sabatier F, Arnaud L, Bonello L, Al-Massarani G, Paganelli F, Poncelet P, Dignat-George F (2008). CD146-based immunomagnetic enrichment followed by multiparameter flow cytometry: a new approach to counting circulating endothelial cells. J Thromb Haemost.

[12] Rowand JL, Martin G, Doyle GV, Miller MC, Pierce MS, Connelly MC, Rao C, Terstappen LW (2007). Endothelial cells in peripheral blood of healthy subjects and patients with metastatic carcinomas. Cytometry Part A.

[13] Strijbos MH, Gratama JW, Schmitz PI, Rao C, Onstenk W, Doyle GV, Miller MC, de Wit R, Terstappen LW, Sleijfer S (2010). Circulating endothelial cells, circulating tumour cells, tissue factor, endothelin-1 and overall survival in prostate cancer patients treated with docetaxel. Eur J Cancer.

[14] Lee KW, Lip GY, Tayebjee M, Foster W, Blann AD (2005). Circulating endothelial cells, von Willebrand factor, interleukin-6, and prognosis in patients with acute coronary syndromes. Blood.

[15] George F, Brouqui P, Boffa MC, Mutin M, Drancourt M, Brisson C, Raoult D, Sampol J (1993). Demonstration of Rickettsia conorii-induced endothelial injury in vivo by measuring circulating endothelial cells, thrombomodulin, and von Willebrand factor in patients with Mediterranean spotted fever. Blood.

[16] Grefte A, van der Giessen M, van Son W, The TH (1993). Circulating cytomegalovirus (CMV)-infected endothelial cells in patients with an active CMV infection. J Infect Dis.

[17] Percivalle E, Revello MG, Vago L, Morini F, Gerna G (1993). Circulating endothelial giant cells permissive for human cytomegalovirus (HCMV) are detected in disseminated HCMV infections with organ involvement. J Clin Invest.

[18] Rodriguez-Ayala E, Yao Q, Holmen C, Lindholm B, Sumitran-Holgersson S, Stenvinkel P (2006). Imbalance between detached circulating endothelial cells and endothelial progenitor cells in chronic kidney disease. Blood Purif.

[19] Mancuso P, Bertolini F (2010). Circulating endothelial cells as biomarkers in clinical oncology. Microvasc Res.

[20] Asicioglu E, Gogas Yavuz D, Koc M, Ozben B, Yazici D, Deyneli O, Akalin S (2010). Circulating endothelial cells are elevated in patients with type 1 diabetes mellitus. Eur J Endocrinol.

[21] McClung JA, Naseer N, Saleem M, Rossi GP, Weiss MB, Abraham NG, Kappas A (2005). Circulating endothelial cells are elevated in patients with type 2 diabetes mellitus independently of HbA(1)c. Diabetologia.

[22] Makin AJ, Blann AD, Chung NA, Silverman SH, Lip GY (2004). Assessment of endothelial damage in atherosclerotic vascular disease by quantification of circulating endothelial cells. Relationship with von Willebrand factor and tissue factor. Eur Heart J.

[23] Woywodt A, Streiber F, de Groot K, Regelsberger H, Haller H, Haubitz M (2003). Circulating endothelial cells as markers for ANCA-associated small-vessel vasculitis. Lancet.

[24] Strijbos MH, Landburg PP, Nur E, Teerlink T, Leebeek FW, Rijneveld AW, Biemond BJ, Sleijfer S, Gratama JW, Duits AJ, Schnog JJ (2009). Circulating endothelial cells: a potential parameter of organ damage in sickle cell anemia?. Blood Cells Mol Dis.

[25] Erdbruegger U, Woywodt A, Kirsch T, Haller H, Haubitz M (2006). Circulating endothelial cells as a prognostic marker in thrombotic microangiopathy. Am J Kidney Dis.

[26] Janssens D, Michiels C, Guillaume G, Cuisinier B, Louagie Y, Remacle J (1999). Increase in circulating endothelial cells in patients with primary chronic venous insufficiency: protective effect of Ginkor Fort in a randomized double-blind, placebo-controlled clinical trial. J Cardiovasc Pharmacol.

[27] Woywodt A, Scheer J, Hambach L, Buchholz S, Ganser A, Haller H, Hertenstein B, Haubitz M (2004). Circulating endothelial cells as a marker of endothelial damage in allogeneic hematopoietic stem cell transplantation. Blood.

[28] Li C, Wu Q, Liu B, Yao Y, Chen Y, Zhang H, Wang C, Cao J, Ge S (2013). Detection and validation of circulating endothelial cells, a blood-based diagnostic marker of acute myocardial infarction. PLoS One.

[29] Damani S, Bacconi A, Libiger O, Chourasia AH, Serry R, Gollapudi R, Goldberg R, Rapeport K, Haaser S, Topol S, Knowlton S, Bethel K, Kuhn P, Wood M, Carragher B, Schork NJ, Jiang J, Rao C, Connelly M, Fowler VM, Topol EJ (2012). Characterization of circulating endothelial cells in acute myocardial infarction. Sci Transl Med.

[30] Kirklin JK, Westaby S, Blackstone EH, Kirklin JW, Chenoweth DE, Pacifico AD (1983). Complement and the damaging effects of cardiopulmonary bypass. J Thorac Cardiovasc Surg.

[31] Schmid FX, Floerchinger B, Vudattu NK, Eissner G, Haubitz M, Holler E, Andreesen R, Birnbaum DE (2006). Direct evidence of endothelial injury during cardiopulmonary bypass by demonstration of circulating endothelial cells. Perfusion.

[32] Skrabal CA, Choi YH, Kaminski A, Steiner M, Kundt G, Steinhoff G, Liebold A (2006). Circulating endothelial cells demonstrate an attenuation of endothelial damage by minimizing the extracorporeal circulation. J Thorac Cardiovasc Surg.

[33] Wittwer T, Choi YH, Neef K, Schink M, Sabashnikov A, Wahlers T (2011). Off-pump or minimized on-pump coronary surgery--initial experience with Circulating Endothelial Cells (CEC) as a supersensitive marker of tissue damage. J Cardiothorac Surg.

[34] Fusari M, Bona V, Muratori M, Salvi L, Salis S, Tamborini G, Biglioli P (2012). Transcatheter vs. surgical aortic valve replacement: a retrospective analysis assessing clinical effectiveness and safety. J Cardiovasc Med (Hagerstown).

[35] Vahanian A, Alfieri O, Al-Attar N, Antunes M, Bax J, Cormier B, Cribier A, De Jaegere P, Fournial G, Kappetein AP, Kovac J, Ludgate S, Maisano F, Moat N, Mohr F, Nataf P, Pierard L, Pomar JL, Schofer J, Tornos P, Tuzcu M, van Hout B, Von Segesser LK, Walther T (2008). Transcatheter valve implantation for patients with aortic stenosis: a position statement from the European association of cardio-thoracic surgery (EACTS) and the European Society of Cardiology (ESC), in collaboration with the European Association of Percutaneous Cardiovascular Interventions (EAPCI). EuroIntervention.

[36] Bertolini F, Shaked Y, Mancuso P, Kerbel RS (2006). The multifaceted circulating endothelial cell in cancer: towards marker and target identification. Nat Rev Cancer.

[37] Kraan J, Strijbos MH, Sieuwerts AM, Foekens JA, den Bakker MA, Verhoef C, Sleijfer S, Gratama JW (2012). A new approach for rapid and reliable enumeration of circulating endothelial cells in patients. J Thromb Haemost.

[38] Boos CJ, Balakrishnan B, Blann AD, Lip GY (2008). The relationship of circulating endothelial cells to plasma indices of endothelial damage/dysfunction and apoptosis in acute coronary syndromes: implications for prognosis. J Thromb Haemost.

[39] Furstenberger G, von Moos R, Lucas R, Thurlimann B, Senn HJ, Hamacher J, Boneberg EM (2006). Circulating endothelial cells and angiogenic serum factors during neoadjuvant chemotherapy of primary breast cancer. Br J Cancer.

[40] Goon PK, Boos CJ, Stonelake PS, Blann AD, Lip GY (2006). Detection and quantification of mature circulating endothelial cells using flow cytometry and immunomagnetic beads: a methodological comparison. Thromb Haemost.

[41] Mancuso P, Antoniotti P, Quarna J, Calleri A, Rabascio C, Tacchetti C, Braidotti P, Wu HK, Zurita AJ, Saronni L, Cheng JB, Shalinsky DR, Heymach JV, Bertolini F (2009). Validation of a standardized method for enumerating circulating endothelial cells and progenitors: flow cytometry and molecular and ultrastructural analyses. Clin Cancer Res.

[42] Elshal MF, Khan SS, Takahashi Y, Solomon MA, McCoy JP (2005). CD146 (Mel-CAM), an adhesion marker of endothelial cells, is a novel marker of lymphocyte subset activation in normal peripheral blood. Blood.

[43] Mancuso P, Burlini A, Pruneri G, Goldhirsch A, Martinelli G, Bertolini F (2001). Resting and activated endothelial cells are increased in the peripheral blood of cancer patients. Blood.

[44] Strijbos MH, Kraan J, den Bakker MA, Lambrecht BN, Sleijfer S, Gratama JW (2007). Cells meeting our immunophenotypic criteria of endothelial cells are large platelets. Cytometry B Clin Cytom.

[45] Mutin M, Canavy I, Blann A, Bory M, Sampol J, Dignat-George F (1999). Direct evidence of endothelial injury in acute myocardial infarction and unstable angina by demonstration of circulating endothelial cells. Blood.

[46] Doerr F, Heldwein MB, Bayer O, Sabashnikov A, Weymann A, Dohmen PM, Wahlers T, Hekmat K (2015). Combination of European System for Cardiac Operative Risk Evaluation (EuroSCORE) and Cardiac Surgery Score (CASUS) to Improve Outcome Prediction in Cardiac Surgery. Med Sci Monit Basic Res.

